# Axial Loading during MRI Induces Lumbar Foraminal Area Changes and Has the Potential to Improve Diagnostics of Nerve Root Compromise

**DOI:** 10.3390/jcm11082122

**Published:** 2022-04-11

**Authors:** Hanna Hebelka, Niklas Rydberg, John Hutchins, Kerstin Lagerstrand, Helena Brisby

**Affiliations:** 1 Institute of Clinical Sciences, Sahlgrenska Academy, University of Gothenburg, SE413 45 Gothenburg, Sweden; hanna.hebelka@vgregion.se (H.H.); niklas.rydberg@vgregion.se (N.R.); john.hutchins@vgregion.se (J.H.); kerstin.lagerstrand@vgregion.se (K.L.); 2 Department of Radiology, Sahlgrenska University Hospital, SE413 45 Gothenburg, Sweden; 3 Department of Orthopaedics, Sahlgrenska University Hospital, SE413 45 Gothenburg, Sweden; 4 Department of Radiation Physics, Sahlgrenska University Hospital, SE413 45 Gothenburg, Sweden

**Keywords:** lumbar foraminal area, axial loading during MRI, lumbar foraminal stenosis, diagnostics

## Abstract

Lumbar foraminal stenosis is a common cause of lumbar radiculopathy and conventionally assessed with magnetic resonance imaging (MRI) in supine-positioned patients. An MRI acquired during spine loading may unmask pathology not otherwise revealed in a relaxed position. Therefore, we investigated how spine loading during MRI affects lumbar foramina. In 89 low-back pain patients’ lumbar, MRIs were performed in a relaxed supine position and during axial loading using a Dynawell^®^ compression device. The smallest area of all intervertebral foramina at levels L3/L4–L5/S1 (534 foramina) was determined using a freehand polygonal tool in parasagittal T2-weighted sequences. The grading system described by Lee et al. was also used to qualitatively assess foraminal stenosis. Overall, a mean reduction of 2.2% (mean −0.89 cm^2^ and −0.87 cm^2^, respectively) was observed (*p* = 0.002), however for individual foramina large variations, with up to about 50% increase or decrease, were seen. Stratified for lumbar level, an area reduction was found for L3/L4 and L4/L5 foramina (mean change −0.03 cm^2^; *p* = 0.036; and −0.03 cm^2^; *p* = 0.004, respectively) but not for L5/S1. When comparing the measured area changes to qualitative foraminal grading, 22% of the foramina with a measured area decrease were evaluated with a higher grading. Thus, detailed information on foraminal appearance and nerve root affection can be obtained using this method.

## 1. Introduction

Lumbar foraminal stenosis is a common cause of leg radiculopathy with an 8–11% prevalence, more often observed in middle-aged or older individuals [1,2]. Degenerative changes in the lumbar spine are the basis for developing lumbar stenosis, central, lateral (recess), and strict foraminal canal stenosis. When an intervertebral disc degenerates, its height gradually decreases, causing the superior articular process of the inferior vertebrae to dislocate forward and upward. Consequently, the volume of the intervertebral foramina at this disc level reduces, the nerve root leaving the spinal canal may be affected, and the compression subsequently may cause radiating pain. An accurate diagnosis of foraminal stenosis forms the basis for improved decision-making regarding the best treatment for such patients.

Magnetic resonance imaging (MRI) is conventionally used to assess potential foraminal stenosis in the supine position. This body position may reduce the diagnostic accuracy since symptoms of radiculopathy are mainly provoked in a standing position. An MRI performed during spinal loading has been shown to unmask the severity of pathology otherwise not revealed in a relaxed supine position, such as the degree or grade of spondylolisthesis, nerve compression caused by disc herniations, and/or severity of central spinal stenosis [3]. Furthermore, specific pathologies not detected in an unloaded position may appear with loading, e.g., facet joint cysts [4,5]. The loading of the lumbar spine may influence foramina differently based on several factors, e.g., spinal segment level, disc angle, loading direction, and the degree of pathological changes [6,7,8,9]. However, despite reports on the influence of spinal loading on intervertebral foramina, no systematic work has evaluated the extent to which the intervertebral lumbar foramina are affected during axial loading of the lumbar spine.

Our study aimed to investigate the influence of spinal loading on lower-level lumbar foramina in a population with unspecific low back pain (LBP) by comparing supine MR images with and without axial loading of the spine.

## 2. Materials and Methods

### 2.1. Participants

A total of 98 patients were consecutively recruited in a 2-year period for an ongoing study examining the impact of lumbar spinal loading in patients with unspecific LBP on different spinal structures by comparing MR images acquired with and without spinal loading. Inclusion criteria for the patients were: age between 20 and 70 years and chronic non-specific LBP, defined as pain with a duration of at least 3 months. Patients were excluded from the study if they reported sciatica symptoms, presented with claustrophobia, had previous back surgery, or if clinical examination indicated a nerve root affection. In addition, adequate sagittal MR images were necessary for the patient to be included in the present sub-study, both with and without axial loading of the spine. Overall, 89 of the initially recruited patients fulfilled all these criteria. The mean age was 43 years (range 27–66 years), including 56 men and 33 women.

The study was approved by the Regional Ethics Review Board and conducted in accordance with the Helsinki Declaration; informed consent was provided and signed by all the patients included.

### 2.2. Magnetic Resonance Imaging

All patients underwent MRI examination (3T scanner/Signa, GE Healthcare, Chicago, IL, USA) of the lumbar spine, performed first in a relaxed supine position (without loading) and immediately thereafter during axial loading in a supine position (with loading) using identical scan parameters. Sagittal T1-weighted (w) sequences (repetition time [TR] 573 ms/echo time [TE] 7.7 ms/slice thickness 3.5 mm) and T2w sequences were performed.

**Figure 1:**
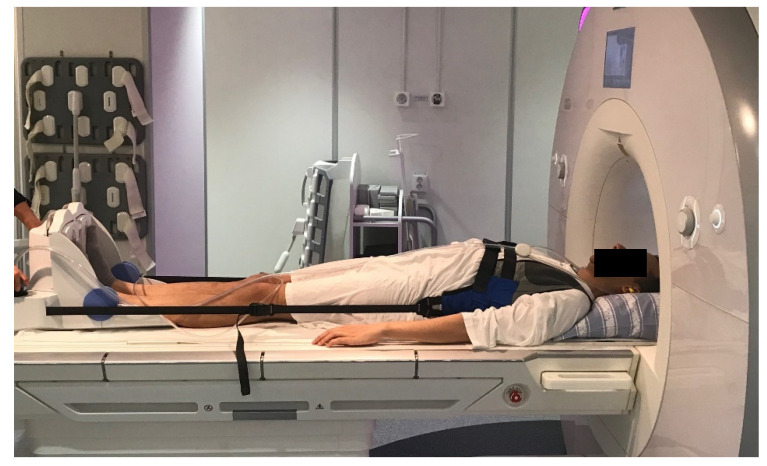
A patient positioned in the Dynawell^®^ compression device just about to undergo magnetic resonance imaging (MRI) examination.

The MRI with loading was performed using a Dynawell^®^, (Dynawell diagnostics Inc., Henderson, NY, USA) compression device. Axial loading of about 50% of the patient’s body weight was applied using a footplate attached to the patient’s harness by side straps. Thus, a position simulating the loading of the lumbar spine in an upright position was achieved using this equipment in the MRI scanner [4,10,11], (See Figure 1). A small cushion was placed beneath the lumbar spine to prevent flexion of the spine during compression.

### 2.3. Evaluation of MRI Investigations

**Figure 2:**
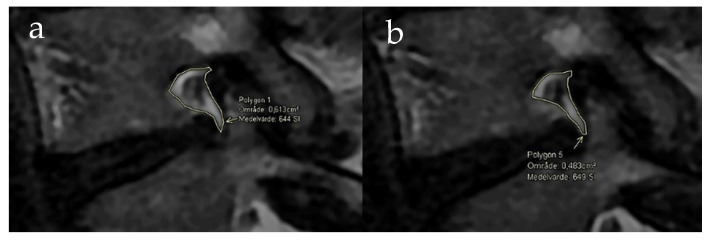
MRI of a right L4–5 foramina without loading of the spine (**a**) and MRI of the same foramina acquired with axial loading (**b**). The measurement area is marked in both images and a reduction in the area was measured. The fat surrounding the nerve root is visually reduced in the MRI with loading.

The intervertebral foramina were evaluated quantitatively and qualitatively; the estimates were compared between MRIs with and without axial loading of the spine. Quantitative evaluation of the intervertebral foramina was performed by measuring the area of the intervertebral foramina L3/L4-L5/S1 bilaterally (534 foramina in total) for sagittal T2w sequences (for each foramina the sagittal slice with the smallest area for both images, with and without load, was selected) using a freehand polygonal tool (See Figure 2). The picture archiving and communication system (PACS) used was AGFA HealthCare Enterprise Imaging (version 8.1.4.112) (AGFA Gaevert Group, Mortsel, Belgium). If the foramen was imaged in more than one sagittal image, the image with the smallest foraminal area was used.

Qualitative assessment of the intervertebral foramina was based on the grading system (grade 0–3) described by Lee et al. [12], where grade 0 refers to the absence of stenosis, and grade 3 represents severe stenosis. The sagittal T1w images were used for this grading. Differences in the intervertebral disc height (dorsal edge) and intervertebral disc angle between MRIs with and without spinal loading sequences, assessed by T1w images, were also determined. In addition, the degeneration grade of the intervertebral discs (L3/4–L5/S1) was assessed according to the Pfirrmann grading system using T2w images without spinal loading [13].

A radiology resident performed blind measurements on all patients after supervised training (by a senior radiologist) on individuals not included in the current study. The resident also performed repeated measurements on 10 patients (60 foramina in total), and the senior radiologist also measured these 10 patients to obtain inter-observer and intra-observer variability. Initially, all measurements were based on MR images acquired without loading. After approximately one month, the MR images acquired during axial loading of the spine were evaluated without access to the initial measurements or images.

### 2.4. Statistics

Descriptive statistics were reported as mean and standard deviation (SD). The comparison between unloaded and loaded foramina measurements was performed with paired t-tests. All the tests were two-sided, and the significance level was set at <0.005. The intra-class correlation (ICC) coefficient was calculated to evaluate the intra- and inter-observer test–retest reliability (absolute agreement, two-way random effects, and single model) regarding continuous measures. Cicchetti’s scheme (1994) was used to interpret the coefficients: <0.40 indicates poor reliability, 0.40–0.59 indicates fair reliability, 0.60–0.74 indicates good reliability, and 0.75–1 indicates excellent reliability. Cohen’s Kappa statistics were used with Landis’ scheme (1977) to evaluate the test–retest for categorical variables, where a Kappa >0.75 was considered a substantial agreement. All analyses used SPSS software (IBM Corp. Released 2020. IBM SPSS Statistics for Windows, Version 27.0. Armonk, NY, USA: IBM Corp.).

## 3. Results

### 3.1. Disc Degeneration Degree in Examined Levels

Pfirrmann grading for the discs was distributed as follows: grade 2 (*n* = 67), grade 3 (*n* = 89), grade 4 (*n* = 87), and grade 5 (*n* = 24).

### 3.2. Foraminal Area Measurements

**Figure 3:**
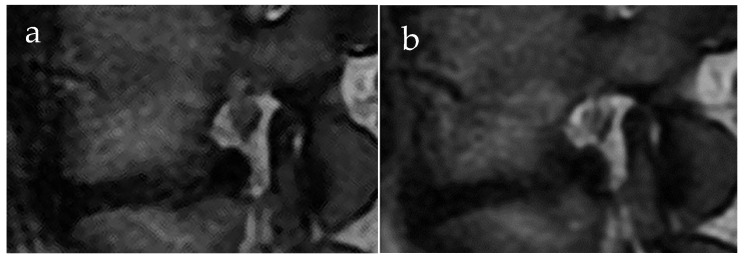
MRI of a left L4–L5 foramina acquired without loading of the spine (**a**) and with loading (**b**). A change in the foraminal shape and increase in disc bulging can be observed; the nerve root is surrounded by fat in both MR images.

Overall, the foraminal area between MR images acquired without and with spinal loading demonstrated a mean reduction of 2.2% (mean 0.89 cm^2^ and 0.87 cm^2^, respectively; *p* = 0.002). A large variation in load-induced changes for the foraminal area was seen (Figure 2 and Figure 3), ranging from a 58% increase to a decrease of 42%, for details see Table 1. When stratified for lumbar level, the area during loading was reduced at the group level for L3/L4 and L4/L5 foramina (mean change −0.03 cm^2^; *p* = 0.036 and −0.03 cm^2^; *p* = 0.004, respectively) but not for L5/S1 (Table 2).

### 3.3. Qualitative Foraminal Evaluation and Conformity to Foraminal Area Change

The number of foramina within each grade according to the Lee classification system was 448 (grade 0), 77 (grade 1), 8 (grade 2), and 1 (grade 3), respectively. Overall, a significant difference was seen between the qualitative grading of foramina on MRI acquired without and with loading (*p* < 0.001). In 133/534 (25%) of the foramina, a change in the foraminal stenosis grade (higher or lower grade) occurred in MRI acquired with spinal loading; a higher grading (narrower foramina) was determined in 102 (19%) for all foramina (*p* < 0.001). When comparing the measured area changes (without/with loading) with the qualitative grading of the foramina, 22% of the foramina with a measured area decrease were evaluated as having a higher grading (area change mean −0.09 cm^2^ SD 0.08), whereas 72% of the foramina with a measured area decrease were evaluated as having the same grade (area change mean −0.14 cm^2^ SD 0.11).

### 3.4. Reliability Measures

For the foraminal area, inter-observer ICC was 0.76 (95% CI 0.62–0.85), and 0.96 for intra-observer, (95% CI 0.93–0.97), both indicating excellent reliability.

## 4. Discussion

The results from the present study demonstrated a significant area decrease in lumbar foramina on group level, in a population with varying degree of disc degeneration and no sciatic symptoms, when comparing MR images acquired during axial loading of the lumbar spine to images acquired without axial loading. However, for individual foramina a large variation of load-induced area change was found with variations of up to about 50%, either as an increase or a decrease. Further, at the levels L3–4 and L4–5, loading induced an area decrease at group level, in opposite to an increase for the L5–S1 level.

In clinical practice, it is not rare to see patients with radiating leg pain, especially if intermittent, where a lumbar MRI indicates no clear explanatory pathology. Furthermore, several spinal levels are sometimes suspected as potential pain sources, and narrowing down where the pathology derives from is desired. A method sometimes used to improve diagnostics is selective nerve root blocks; however, the specificity is relatively low [14]. Therefore, MRI investigations revealing dynamic foraminal changes can play a role in improving diagnostics, and the non-invasiveness is appealing. Only images in an unloaded position can be obtained during a conventional lumbar spine MRI investigation. A loaded spine position, and perhaps more relevant—the comparison between MRI taken with the spine in an unloaded and loaded position—may add information regarding nerve root compromise occurring or becoming aggravated in a loaded position. The foraminal area changes in the present study were expected based on the anatomy and shape of the lumbar spine. The area changes were relatively large and demonstrated that large variations between segments could detect clinically relevant changes since similar changes also likely occur in patients with radiculopathy. This observation was strengthened by the finding that about 20% of the foramina with a load-induced measured area decrease in this group of patients without any sciatic symptoms, was evaluated having a higher qualitative grading. Overall, these findings advocate the potential use of the present method; however, it requires further testing on patients with unclear leg pain symptoms. The potential clinical value is supported by a study on patients with spondylolistheses, where a change in olisthesis with loading (dynamic component) was demonstrated to correlate not only to a decrease in dural sac area, but also with worsening of patient symptoms [15].

There are many factors, alone or in combination, that may affect specific foramen during the loading of the spine, e.g., disc degeneration degree in the actual level, the direction of the loading forces applied, disc height, disc angle [3,6,7,8], and other potential factors, such as muscle tension. The effect of a specific loading situation on a single foramen both in real life (standing/sitting) and in a loaded supine position, particularly during an MRI, is difficult to predict. However, MRI data of the spine during loading can help us better understand the dynamics of the foramina and increase overall knowledge on spine dynamics, providing better interpretations also of unloaded MRI investigations.

When comparing images with/without loading, the differences seen between spinal levels may suggest that underestimating a nerve root compromise is higher at the L3–L4 and L4–L5 foramina than at the L5–S1 level. In experimental set-ups and clinical studies, a spinal extension has been shown to decrease foraminal height, width, and area [6,16]; it also has less of an effect on the L5–S1 levels [8]. However, the observed differences between levels may be caused by different factors, such as the direction of the loading forces and/or degenerative factors.

Examining the human body during loading can be performed in other ways than described here. Using the upright MRI technique when patients sit or stand during image capturing appears like the optimal investigation and has also been suggested [15]. A few reports on the foraminal area using upright MRI also indicate a decrease in the lumbar foraminal area at standing [17,18]; however, this methodology has not reported reliable measurements. The provocation of sciatic pain during an MRI examination decreases a patient’s ability to remain still, with a higher risk expected for motion artifacts and decreased accuracy for an upright/standing MRI examination than if performed in a supine position using a loading device. Further, the access to upright MRI is limited at most centra. Another technique that addresses foraminal changes in standing uses dynamic digital tomosynthesis-radiculography, which includes ionizing radiation [19,20].

The major strength of our present study was the relatively large number of individuals with varying grades of disc degeneration, and all imaged with and without axial loading of the spine. Nonetheless, the number of patients/number of foramina do not allow sub-analysis regarding possible different influencing factors, such as disc degeneration degree, which is, of course, a limitation. Another limitation is that conventional sagittal two-dimensional (2D) MRI sequences were used. The superiority of 3D sequences in diagnostic performance regarding foraminal nerve root affection has been reported [21]. The 3D sequences enable the acquisition of images perpendicular to each foramen, with the ability to visualize the nerve root in each plane. The use of parasagittal 2D sequences to evaluate the foraminal area is accompanied by inherent limitations and do not provide a complete picture of how the foramina change shape or volume with loading. However, the high intraclass correlation coefficient values for foraminal area measurement and good interobserver agreement support the reliability of the measurement technique.

## 5. Conclusions

In conclusion, detailed information on foraminal appearance and nerve root affection can be obtained using MRIs acquired with and without loading of the spine. As a next step, it would be desirable to examine a group of patients with intermittent nerve root symptoms and observe whether their symptoms are linked to changes detected by MRIs acquired with and without spinal load. It is likely that MRIs performed with spinal loading unmasks nerve root affection not seen in an unloaded position and may have the potential to improve diagnostics, especially when conventional MRI findings do not match the clinical symptoms of lumbar radiculopathy. Increased knowledge of degenerative spine dynamics may improve diagnostics and thereby patient selection for various treatments.

## Figures and Tables

**Table 1:** Distribution of foraminal area change at MRI during loading compared to unloaded.

Area Change with Loading	Foramina (*n* = 534) *n* (%)
Decrease	265 (50%)
0–10%	128 (24%)
10–20%	79 (15%)
20–30%	43 (8%)
30–40%	13 (2%)
>40%	2 (0.4%)
Increase	222 (42%)
0–10%	108 (20%)
10–20%	51 (10%)
20–30%	36 (7%)
30–40%	16 (3%)
>40%	11 (2%)
Unchanged	47 (9%)

**Table 2:** Comparison of the foraminal area with and without axial spinal loading for all measured foramina, stratified for lumbar level.

	Without Axial Load (cm^2^) *	With Axial Load (cm^2^) *	Paired Differences (cm^2^) *	*p*-Value
All foramina *n* = 534	0.89 (0.27)	0.87 (0.26))	−0.02 (0.15)	0.002
Levels				
L3/L4	0.99 (0.29)	0.96 (0.28)	−0.03 (0.17)	0.036
L4/L5	0.80 (0.20)	0.78 (0.20)	−0.03 (0.11)	0.004
L5/S1	0.87 (0.27)	0.86 (0.25)	−0.008 (0.15)	0.47

* Values are mean and (SD).

## Data Availability

The measurement data details are available upon request from the corresponding author. The MRI data are not publicly available due to ethical and privacy policy reasons.
